# Comparison between myocardial function assessed by echocardiography during hospitalization for COVID-19 and at 4 months follow-up

**DOI:** 10.1007/s10554-021-02346-5

**Published:** 2021-07-20

**Authors:** F. M. A. van den Heuvel, J. L. Vos, B. van Bakel, A. L. Duijnhouwer, A. P. J. van Dijk, A. C. Dimitriu-Leen, P. C. Koopmans, Q. de Mast, F. L. van de Veerdonk, F. H. Bosch, B. van den Borst, T. M. H. Eijsvogels, R. R. J. van Kimmenade, R. Nijveldt

**Affiliations:** 1 Department of Cardiology, Radboud University Medical Center, Geert Grooteplein Zuid 10, 6525 GA Nijmegen, The Netherlands; 2 Department of Physiology, Radboud Institute for Health Sciences, Radboud University Medical Center, Nijmegen, The Netherlands; 3 Section Biostatistics, Department of Health Evidence, Radboud University Medical Center, Nijmegen, The Netherlands; 4 Department of Internal Medicine and Radboud Center for Infectious Diseases, Radboud University Medical Center, Nijmegen, The Netherlands; 5 Department of Internal Medicine, Radboud University Medical Center, Nijmegen, The Netherlands; 6 Department of Pulmonology, Radboud University Medical Center, Nijmegen, The Netherlands

**Keywords:** COVID-19, SARS-CoV-2, Echocardiography, Myocardial function

## Abstract

In patients hospitalized for corona virus infectious disease 19 (COVID-19) it is currently unknown whether myocardial function changes after recovery and whether this is related to elevated cardiac biomarkers. In this single center, prospective cohort study we consecutively enrolled hospitalized COVID-19 patients between 1 April and 12 May 2020. All patients underwent transthoracic echocardiography (TTE) evaluation during hospitalization and at a median of 131 days (IQR; 116–136) follow-up. Of the 51 patients included at baseline, 40 (age: 62 years (IQR; 54–68), 78% male) were available for follow-up TTE. At baseline, 68% of the patients had a normal TTE, regarding left ventricular (LV) and right ventricular (RV) volumes and function, compared to 83% at follow-up (p = 0.07). Median LV ejection fraction (60% vs. 58%, p = 0.54) and tricuspid annular plane systolic excursion (23 vs 22 mm, p = 0.18) were comparable between hospitalization and follow-up, but a significantly lower RV diameter (39 vs. 34 mm, p = 0.002) and trend towards better global longitudinal strain (GLS) (− 18.5% vs − 19.1%, p = 0.07) was found at follow-up. Subgroup analysis showed no relation between patients with and without elevated TroponinT and/or NT-proBNP during hospitalization and myocardial function at follow-up. Although there were no significant differences in individual myocardial function parameters at 4 months follow-up compared to hospitalisation for COVID-19, there was an overall trend towards normalization in myocardial function, predominantly due to a higher rate of normal GLS at follow-up.

**Supplementary Information:**

The online version contains supplementary material available at 10.1007/s10554-021-02346-5.

## What is new?

- In recovered COVID-19 patients there is a trend towards better global longitudinal strain and the combination of echocardiographic parameters for LV and RV volumes and function 4 months after hospitalization.
- Elevated TroponinT and/or NT-proBNP during hospitalization was not associated with myocardial function at follow-up.
- Most recovered COVID-19 patients have normal myocardial function on echocardiography at 4 months follow-up (83%).

## Introduction

Corona virus infectious disease 19 (COVID-19) has a huge impact on the global healthcare system. A substantial percentage of COVID-19 patients requires hospitalization for supplemental oxygen or invasive ventilation [1]. In hospitalized COVID-19 patients, the reported cardiovascular complications are predominantly acute myocardial injury [2, 3], venous thrombo-embolic events [4, 5] and arrhythmia [5]. Myocardial function assessed by echocardiography, indicated that left ventricular (LV) systolic dysfunction occurred in 10–27%, and right ventricular (RV) systolic dysfunction in 10–39% of non-selected hospitalized COVID-19 patients [6, 7]. In hospitalized COVID-19 patients both structural cardiac abnormalities [8, 9] and acute myocardial injury, defined as elevated TroponinT [10], are linked to in-hospital mortality [2, 3]. However, elevated cardiac biomarkers TroponinT and NT-proBNP do not seem to be related to myocardial dysfunction in hospitalized COVID-19 patients [6]. It is currently unknown whether myocardial function changes over time in recovered COVID-19 patients and whether this is related to elevated cardiac biomarkers during hospitalization. Therefore, the aim of this study is to evaluate longitudinal changes in myocardial function in patients hospitalized with COVID-19 compared to 4 months after discharge. Additionally, the relation between elevated cardiac biomarkers during hospitalization and myocardial function at follow-up was evaluated.

## Methods

### Patient population

In this single center prospective cohort study, we recruited non-selected, previously hospitalized COVID-19 patients [6]. In short, non-selected and consecutively admitted patients hospitalized at the COVID-19 nursing ward of the Radboudumc (Nijmegen, the Netherlands) were included between 1 April and 12 May, 2020. Polymerase chain reaction (PCR) testing of a nasopharyngeal sample and a non-enhanced low dose CT thorax was performed to confirm the diagnosis of COVID-19. Further information about in- and exclusion criteria were described previously [6]. Patient data, including demographics, medical history, diagnostics, laboratory examinations, treatment, cardiovascular complications and outcomes were collected and analyzed. Follow-up data were derived from our local electronic health record system. All patients received an online questionnaire for assessing self-reported new and persisting symptoms after COVID-19, medical procedures/surgery, out-patient visits and rehospitalization. The study protocol was approved by the local medical ethics committee (nr. 2020‐6765) and written informed consent was obtained from all patients.

### Echocardiographic assessment

All patients underwent a first clinical transthoracic echocardiogram (TTE) at the COVID-19 nursing ward, in supine position, and a second follow-up TTE (median 131 days, IQR; 116–136) afterwards at the outpatient clinic. TTE was focused on LV- and RV systolic function, LV diastolic function and global longitudinal strain (GLS). We did not extensively assess valvular function. All TTE’s were performed by experienced sonographers using one single ultrasound system (Affiniti 70 General, Philips Healthcare, Best, the Netherlands) in hospitalized patients, and another single ultrasound system (EPIQ 7, Philips Healthcare, Best, the Netherlands) at the outpatient clinic. Offline analysis was performed by one single investigator (EACVI TTE certified) using dedicated software (AGFA Enterprise Imaging Cardiology version 8.1.2, AGFA HealthCare, Mortsel, Belgium). Detailed information about echocardiographic assessment was described previously [6]. In short, LV function was assessed with LV ejection fraction (LVEF), GLS and the ratio between early mitral inflow velocity and mitral annular early diastolic velocity (E/e′). GLS was measured using speckle tracking echocardiography on a three beats acquisition from three apical long-axis views with a frame rate > 60 frames/s. The average three plane GLS was used. LV dysfunction was defined as LVEF below 52% [11] and/or GLS worse than − 18% [12] and/or E/e′ > ratio 14. RV function was assessed with tricuspid annular plane systolic excursion (TAPSE) and RV systolic excursion velocity (RV S’). RV dysfunction was defined as a TAPSE < 17 mm [11] and/or a RV S’ < 10 cm/s [11]. For measuring LVEF, preferably a triplane LVEF was used. In case of poor image quality, Simpson’s biplane LVEF was used next, and otherwise eyeball LVEF assessment [11]. The best method for quantifying LVEF of each TTE is used for further analysis. A 3 dimensional (3D) LVEF using Dynamic Heart Model (Philips Healthcare, Best, the Netherlands) was only available at follow-up. We compared LVEF between the two TTE’s using the same method for quantifying LVEF preferably with triplane and otherwise with Simpson’s biplane. If only eyeball LVEF was available in one of the TTE’s, we compared LVEF with the average LVEF assessed by other quantifying methods including 3D LVEF. We only compared eyeball LVEF if this was the best method of assessment in both TTE’s. A normal TTE regarding LV and RV volumes and function was defined as: LVEF ≥ 52%, GLS ≤ − 18%, TAPSE ≥ 17 mm, RV S’ ≥ 10 cm/s, E/e′ ratio ≤ 14, indexed LVmass ≤ 115 g/m^2^, Indexed LV end diastolic diameter ≤ 31 mm and RV basal diameter ≤ 42 mm [11].

### Cardiac biomarkers

High-sensitive TroponinT and NT-proBNP concentrations (Roche Diagnostics, Penzberg, Germany) were measured during hospitalization, within 48 h of the TTE. Elevation in TroponinT levels were defined as a value > 14 ng/L (i.e. 99th percentile of upper reference limit) and elevated NT-proBNP was defined as a value > 300 pg/mL.

### Statistical analysis

Categorical variables are summarized with counts and percentages, continuous variables with median and interquartile range (non-normal distribution). All statistical analyses were performed using IBM SPSS statistics version 25 (Armonk, New York, United States). A p-value of < 0.05 will be considered significant. The Wilcoxon signed-rank test was used for comparing echocardiographic parameters (non-normal distribution) across timepoints. For categorical variables a McNemar test for paired data is used. Subgroup analyses of echocardiographic parameters at follow-up were performed using the Mann–Whitney U test. The following subgroups were defined: intensive care unit (ICU) admission, pulmonary embolism diagnosed during hospitalization, elevated TroponinT, elevated NT-proBNP and persistent self-reported symptoms.

## Results

### Study population

Fifty-one patients were initially enrolled of which 40 (78%) were available for follow-up: 5 were excluded because of no consent to participate, 3 because of palliative oncology care, 1 because of large travel distance,1 because of hospitalization elsewhere and 1 deceased during index hospitalizing. Of the remaining 40 patients, median age was 62 years (IQR; 54–68), 78% was male, and the median body mass index was 27 kg/m^2^ (IQR; 24–29) (Table 1). Twenty five percent had a history with cardiovascular disease including obstructive coronary artery disease (13%), myocardial infarction (13%) and atrial fibrillation (8%). Hypertension (40%) was the most frequent cardiovascular risk factor, followed by diabetes (18%). Of all patients, 25% was on immunosuppressive therapy, and 13% on oral anticoagulation at admission. The majority of patients (90%) had a positive COVID-19 PCR test. The remaining 10% was considered as ‘probable COVID-19’, based on high clinical suspicion of COVID-19 and CT findings (CO-RADS 4–5), and were treated for COVID-19. Low dose CT thorax was performed in 90% with a median CO-RADS score of 5 and severity score of 13. Duration of hospitalization was 9 days (IQR; 7–22). Nearly all patients received supplemental oxygen (93%) and 14 patients (35%) were admitted to the intensive care unit (ICU), of whom 12 (30%) were intubated and mechanically ventilated mostly in prone position (Table 2). Of the initially 51 patients, only 3 patients were transferred to the ICU department after the first echocardiogram, of whom one patient deceased. Sixteen patients came from the ICU department and underwent echocardiography on the COVID-19 nursing ward thereafter.

**Table 1:** Baseline characteristics

	Patients (n = 40)
Male	31 (77.5%)
Age (years)	62.5 (53.5–68.0)
Body mass index (kg/m^2^)	27 (24–29)
Comorbidities	
*Cardiac history*	n = 10 (25%)
Obstructive coronary artery disease	n = 5 (12.5%)
Myocardial infarction	n = 5 (12.5%)
Non-ischemic cardiomyopathy	n = 0 (0%)
Heart failure	n = 0 (0%)
Atrial fibrillation	n = 3 (7.5%)
Ventricular arrythmias	n = 1 (2.5%)
Moderate- to severe valvular disease	n = 1 (2.5%)
Cardiac surgery	n = 1 (2.5%)
Cardiac electronic device	n = 1 (2.5%)
Hypertension	n = 16 (40%)
Diabetes mellitus	n = 7 (17.5%)
Currently smoking	n = 3 (7.5%)
Cerebrovascular disease	n = 1 (2.5%)
Chronic renal failure (GFR < 30 or dialysis)	n = 1 (2.5%)
Chronic respiratory diseases (COPD/asthma)	n = 5 (12.5%)
Medication at admission	
Immunosuppressive therapy	n = 10 (25%)
Angiotensin converting enzyme inhibitors	n = 6 (15%)
Angiotensin II receptor blockers	n = 4 (10%)
Oral anticoagulation	n = 5 (12.5%)
Diagnosis of COVID-19 infection	
Positive PCR test	n = 36 (90%)
CT-scan performed	n = 38 (95%)
CO-RADS classification based on the CT-scan	n = 36 (90%)
CO-RADS 1	n = 0 (0%)
CO-RADS 2	n = 0 (0%)
CO-RADS 3	n = 1 (2.5%)
CO-RADS 4	n = 3 (7.5%)
CO-RADS 5	n = 22 (55%)
CO-RADS 6	n = 10 (25%)
CT severity score	13 (10.5–16.5)
Laboratory findings at admission, median (IQR)	
Hemoglobin (mmol/L)	8.4 (7.7–9.0)
Leucocytes (10^9^/L)	6.8 (4.9–9.7)
C-reactive protein (mg/L)	88 (48–165)
D-dimer (ng/mL)	1880 (505–2715)
Procalcitonin (μg/L)	0.20 (0.09–1.08)
GFR (mL/min/kg/m^2^)	81 (66–90)
pH	7.47 (7.44–7.49)
Lactate (mmol/L)	1.3 (1.2–1.8)
TroponinT at any timepoint	n = 37 (92.5%)
Troponin (median ng/L)	12 (8–19)
Troponin > 14 ng/L at any time point	n = 19 (47.5%)
Troponin > 50 ng/L at any time point	n = 3 (7.5%)
Troponin > 140 ng/L at any time point	n = 1 (2.5%)
NT-proBNP at any timepoint	n = 38 (95%)
NT-proBNP (median pg/mL)	315 (94–695)
NT-proBNP > 300 pg/mL	n = 20 (50%)
NT-proBNP > 1000 pg/mL	n = 3 (7.5%)

Values are in median and interquartile range, or n (%)

*COPD* chronic obstructive pulmonary disease, *CO-RADS* COVID-19 Reporting and Data System, *CT* computed tomography, *GFR* glomerular filtration rate, *PCR* polymerase chain reaction

**Table 2:** Treatment and outcome of the patients

	Patients (n = 40)
Treatment	
Treatment with nasal canula/face mask	n = 37 (92.5%)
Nasal high flow therapy	n = 6 (15%)
Mechanical ventilation, n(%)	n = 12 (30%)
Number of days (IQR)	16 (11–22)
Prone ventilation	n = 11 (27.5%)
Medium care unit admission	n = 1 (2.5%)
Intensive care unit admission	n = 14 (35%)
Days of admission (IQR)	14 (9–24)
Diagnosed in hospital complications	
Acute heart failure	n = 3 (7.5%)
Type 1 myocardial infarction	n = 0 (0%)
Type 2 myocardial infarction	n = 1 (2.5%)
Myocarditis	n = 0 (0%)
Ventricular arrythmia	n = 0 (0%)
Atrial fibrillation	n = 3 (7.5%)
CVA/TIA	n = 2 (5%)
Pulmonary embolism	n = 7 (17.5%)
Acute kidney failure	n = 3 (7.5%)
Discharge	
Duration of hospital admission (days)	9 (7–22)
Complications after discharge	
Days of follow-up after first TTE (IQR)	200 (191–206)
Pulmonary embolism	n = 2 (%)
Myocardial infarction	n = 0 (0%)
Acute heart failure	n = 0 (0%)
Atrial fibrillation	n = 1 (2.5%)
Myocarditis	n = 0 (0%)
Hospitalization for cardiac cause	n = 0 (0%)
Emergency department visit	n = 1 (2.5%)
Deceased	n = 0 (0%)
Self-reported symptoms after COVID-19*	
Dyspnea	n = 11 (27.5%)
Chest pain	n = 3 (7.5%)
Peripheral edema	n = 3 (7.5%)
Fatigue	n = 7 (17.5%)

Values are in median and interquartile range, or n (%)

*CVA* cerebrovascular accident, *TIA* transient ischaemic attack, *TTE* transthoracic echocardiogram

*symptoms assessed by online questionnaire at 200 days (IQR; 191–206) after TTE

### Echocardiographic findings

**Fig. 1:**
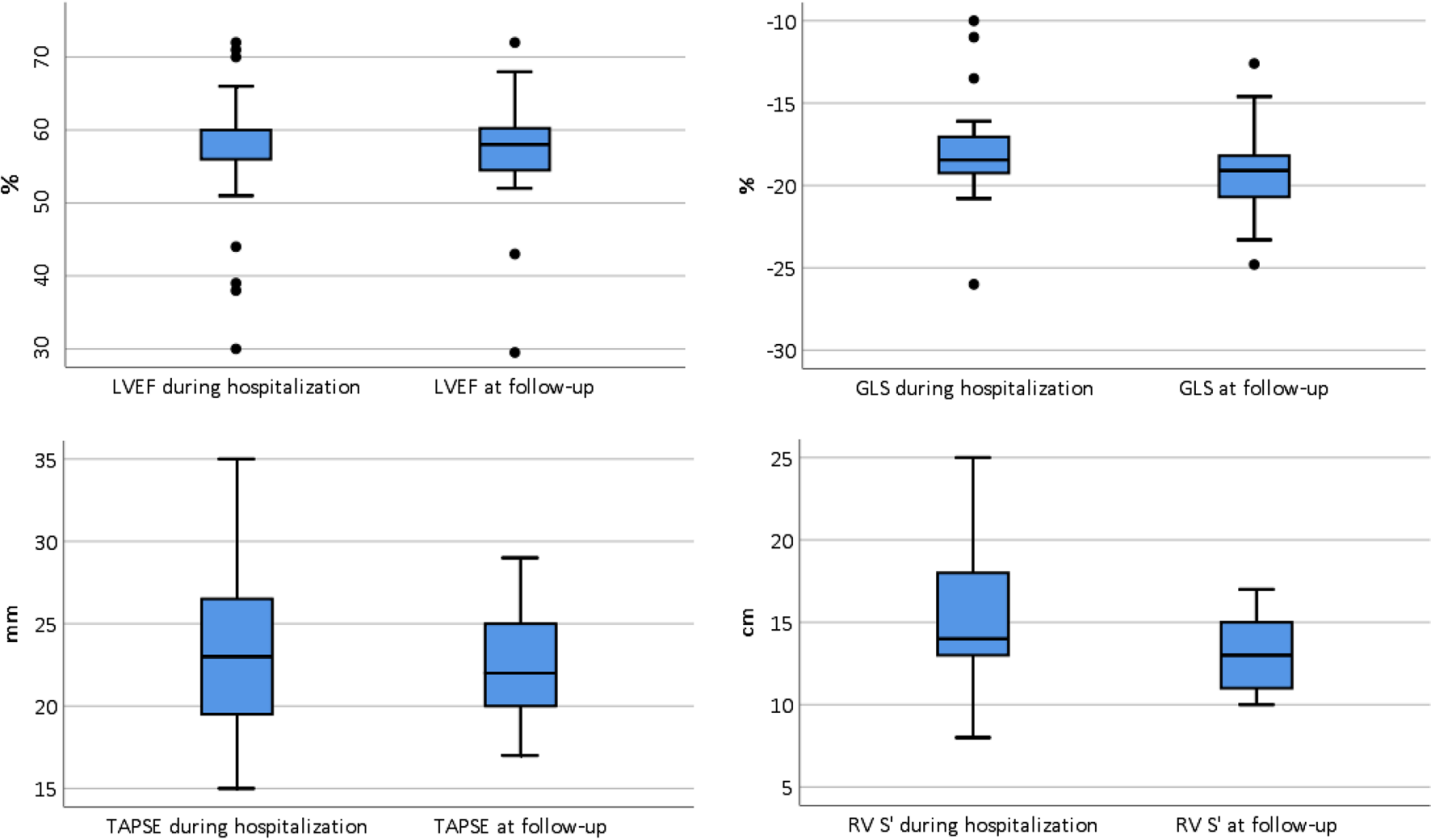
Boxplot of cardiac function parameters. Boxplot of median and interquartile range of left ventricular ejection fraction (LVEF), global longitudinal strain (GLS), right ventricular systolic excursion velocity (RV S’) and tricuspid annular plane systolic (TAPSE) during hospitalization for corona virus infectious disease 19 (COVID-19) and at follow-up

**Fig. 2:**
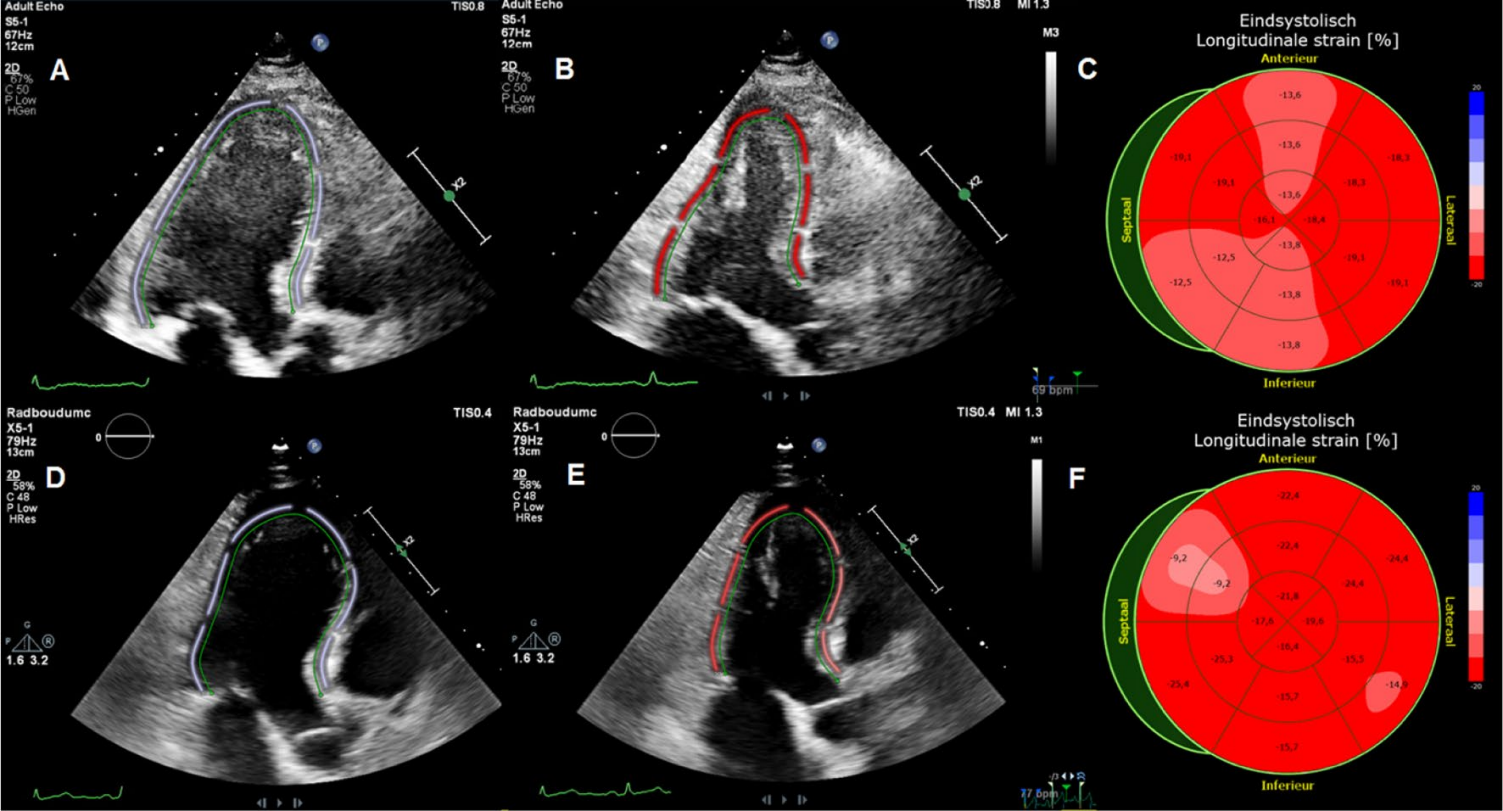
Typical case. Typical case of a patient with abnormal global longitudinal strain during hospitalization (− 16.1%) with normal left ventricular ejection fraction (54%) and normal global longitudinal strain (− 18.8%) and left ventricular ejection fraction (54%) at 4 months follow-up. **A**: apical 3 chamber with end diastolic tracing of left ventricle. **B**: apical 3 chamber with end systolic tracing of left ventricle. **C**: bull’s eye plot of global longitudinal strain. **D**: apical 3 chamber with end diastolic tracing of left ventricle. **E**: apical 3 chamber with end systolic tracing of left ventricle. **F**: bull’s eye plot of global longitudinal strain. Image **A**–**C** are during hospitalization and **D**–**F** at 4 months follow-up

All echocardiographic parameters are summarized in Table 3. Patients underwent transthoracic echocardiography at a median of 4 days (IQR; 2–11 days) after hospitalization and second TTE at a median of 131 days (IQR; 116–136) afterwards. Comparing TTE parameters at hospitalization and follow-up, there were no differences between LVEF (60 (IQR; 56–60) % vs. 58 (IQR; 54–60) %, p = 0.54), LV diastolic function (7.3 vs 7.5 E/e′ ratio, p = 0.90), GLS (− 18.5% vs − 19.1%, p = 0.07) and TAPSE (23 vs 22 mm, p = 0.18). Fore comparing LVEF a triplane EF was used in 20 patients, biplane EF in 5 patients, eyeball EF to average EF in 11 patients and in 4 patients only eyeball EF was used. RV basal diameter (39 vs. 34 mm, p = 0.002) and LV mass (83 vs. 71 g/m^2^, p = 0.04) were higher in patients during hospitalization for COVID-19 compared to follow-up. RV S’ (14 vs.13 cm/s, p = 0.01) was different between both TTE’s although the absolute value of RV S’ at follow-up was in all but 1 patient (3%) within the normal range. During hospitalization, 13% had abnormal LVEF, 38% abnormal GLS, 5% abnormal TAPSE, 6% abnormal RV S’ and 10% abnormal RV basal diameter. At follow-up and compared to hospitalization, 5% had abnormal LVEF (p = 0.25), 18% abnormal GLS (p = 0.13), 0% abnormal TAPSE (p = 0.50), 3% abnormal RV S’ (p = 0.50) and 3% abnormal RV basal diameter (p = 0.50). With regard to a combination of LV and RV volumes and function parameters 67.5% of patients had a normal TTE during hospitalization for COVID-19 and 82.5% at follow-up, with a trend towards statistical significance (P = 0.07). Median LVEF, GLS, TAPSE and RV S’ during hospitalization and at follow-up are shown in Fig. 1. Comparison of all cardiac function parameters during hospitalization and at follow-up in each patient are included in the supplemental data. A typical case is shown in Fig. 2. Subgroup analysis revealed no differences regarding myocardial function at follow-up between patients with and without ICU admission or pulmonary embolism diagnosed during hospitalization (Table 4).

**Table 3:** Echocardiographic parameters of patients

	TTE at baseline N = 40	TTE at follow-up N = 40	p value
LV dimensions, median (IQR)			
Indexed LVEDd (mm/m^2^)	24 (21 to 26)	23 (22 to 26)	0.976
Indexed LV mass (g/m^2^)	83 (68 to 92)	71 (61 to 95)	0.036
LV systolic function median (IQR)			
All EF % (n = 40)	60.0 (55.5 to 60.0)	58.0 (54.3 to 60.4)	0.544
Triplane EF–triplane EF (n = 20)	57 (52.3 to 59.0)	55.0 (53.3 to 58)	0.793
Biplane EF–biplane EF (n = 5)	63.0 (59.5 to 66.0)	61.0 (58.5 to 62.0)	0.221
Eyeball EF–average EF (n = 11)	60.0 (60.0 to 60.0)	60.0 (56.5 to 63.0)	0.475
Eyeball EF–eyeball EF (n = 4)	60.0 (60.0 to 60.0)	60.0 (60.0 to 60.0)	> 0.99
3D EF % (n = 32)	–	61 (55.5 to 64)	–
Global longitudinal strain (%)	n = 21	n = 28	
	− 18.5 (− 19.5 to − 17.0)	− 19.1 (− 20.8 to − 18.2)	0.067
LV diastolic function median (IQR)			
E/e′ ratio	n = 40	n = 40	
	7.3 (6.0 to 9.8)	7.5 (5.9 to 8.8)	0.898
RV dimension and function median (IQR)			
RV basal diameter (mm)	n = 30	n = 33	
	39 (33 to 41)	34 (30 to 38)	0.002
TAPSE (mm)	n = 40	n = 39	
	23 (19 to 27)	22 (20 to 25)	0.181
RV S’ (cm/s)	n = 35	n = 38	
	14 (12 to 18)	13 (11 to 15)	0.01
Other parameters			
Normal LV and RV volumes and function**	67.5%	82.5%	0.07*
Abnormal LVEF (< 52%)	12.5%	5.0%	0.25*
Abnormal GLS (> − 18%)	38.1%	17.9%	0.125*

All values were tested with a Wilcoxon signed-rank test, except for (*) which was done with a McNemar test. Values are in median and interquartile range, or n (%)

*E/e′* early mitral inflow velocity/mitral annular early diastolic velocity, *EF* ejection fraction, *IQR* interquartile range, *LV* left ventricular, *LVEDd* left ventricular end-diastolic dimension, *LVESd* left ventricular end-systolic dimension, *LVEF* LV ejection fraction, *RV* right ventricular, *RV S’* right ventricular systolic excursion velocity, *TAPSE* tricuspid annular plane systolic excursion, *TTE* transthoracic echocardiogram

**Normal TTE was defined as: LVEF ≥ 52%, GLS ≤ − 18%, TAPSE ≥ 17 mm, RV S’ ≥ 10 cm/s, E/e′ ratio ≤ 14, indexed LVmass ≤ 115 g/m^2^, Indexed LVEDd ≤ 31 mm and RV basal diameter ≤ 42 mm

**Table 4:** Subgroup analysis at follow-up

TroponinT	< 14 ng/l (n = 18)	> 14 ng/l (n = 19)	*p* value
LVEF % (median/IQR)	55.5 (54.3 to 59.8)	57.0 (52.3 to 61.3)	0.552
GLS % (median/IQR)	− 19.1 (− 20.7 to − 18.1)	− 19.7 (− 21.4 to − 17.4)	0.593
TAPSE mm (median/IQR)	21.0 (20.0 to 23.8)	23.0 (19.5 to 26.0)	0.621
RV S’ cm/s (median/IQR)	12.0 (10.3 to 15.5)	14 (11.5 to 15.5)	0.525
NT-proBNP	< 300 pg/mL (n = 18)	> 300 pg/mL (n = 20)	
LVEF % (median/IQR)	57.0 (54.0 to 59.5)	55.5 (52.3 to 60.4)	0.557
GLS % (median/IQR)	− 19.7 (− 21.4 to − 18.2)	− 19.1 (− 20.7 to − 17.0)	0.429
TAPSE mm (median/IQR)	21.0 (19.5 to 26.0)	22.5 (20.0 to 23.8)	0.368
RV S’ cm/s (median/IQR)	12.0 (11.0 to 15.0)	13.0 (11.0 to 16.0)	0.886
Treatment	No ICU admission (n = 26)	ICU admission (n = 14)	
LVEF % (median/IQR)	56.0 (53.5 to 60.3)	55.5 (52.5 to 60.3)	0.659
GLS % (median/IQR)	− 19.1 (− 21.1 to − 17.3)	− 19.7 (− 20.5 to− 18.3)	0.981
TAPSE mm (median/IQR)	21.0 ( 20.0 to 24.5)	23 (18.8 to 26.0)	0.871
RV S’ cm/s (median/IQR)	12.0 (11.0 to 15.5)	13.5 (10.5 to 15.8)	0.783
Complication during hospitalization	No pulmonary embolism (n = 33)	Pulmonary embolism (n = 7)	
LVEF % (median/IQR)	58.0 (54.0 to 60.3)	58.0 (55.0 to 61.0)	0.775
GLS % (median/IQR)	− 19.1 (− 21.2 to− 18.0)	− 19.4 (− 20.1 to − 18.6)	0.921
TAPSE mm (median/IQR)	22.0 (20.0 to 25.0)	22.0 (20.0 to 24.0)	0.941
RV S’ cm/s (median/IQR)	13.0 (11.0 to 15.0)	13.0 (11.0 to 15.0)	0.805
Symptoms (dyspnea/chest pain/fatigue/peripheral edema after COVID-19)	No symptoms (n = 26)	Symptoms (n = 14)	
LVEF % (median/IQR)	56.8 (54.0 to 60.1)	59.3 (56.5 to 61.9)	0.151
GLS % (median/IQR)	− 19.1 (− 20.4 to − 18.3)	− 19.1 (− 21.9 to − 17.3)	0.658
TAPSE mm (median/IQR)	22.5 (20.0 to 25.3)	21.0 (19.5 to 25.0)	0.765
RV S’ cm/s (median/IQR)	13.0 (11.0 to 15.0)	14.0 (10.5 to 15.0)	0.938

All values were tested with a Mann–Whitney U test

*GLS* global longitudinal strain, *LV* left ventricular, *LVEF* left ventricular ejection fraction, *RV S’* right ventricular systolic excursion velocity, *TAPSE* tricuspid annular plane systolic excursion

### Cardiac biomarkers

At least one TroponinT value was available in 92.5% of all patients, and 47.5% had elevated TroponinT during hospitalization. NT-proBNP was measured in 95% of all patients, of whom 50% had elevated NT-proBNP during hospitalization. All laboratory values are shown in Table 1. Subgroup analysis showed no differences regarding myocardial function at follow-up between patients with and without elevated TroponinT and/or NT-proBNP (Table 4).

### Outcome

The most frequently diagnosed in-hospital complication was pulmonary embolism (17.5%) followed by atrial fibrillation (7.5%), acute heart failure (7.5%), and acute kidney failure with a glomerular filtration rate below 30 mL/min/1.73 m^2^ (7.5%). Follow-up after discharge was available in all patients, with a median duration of 200 days (IQR; 191–206) after the first TTE. After discharge, 2 (5%) patients had a pulmonary embolism diagnosed on a chest CT which was performed for other reasons and 1 patient (3%) developed de novo atrial fibrillation (Table 2). From the online post COVID-19 questionnaire, 14 patients (35%) had self-reported new and/or persisting symptoms after COVID-19. Dyspnea (28%) was the most frequent symptom, followed by fatigue (18%), chest pain (8%), and peripheral edema (8%). Subgroup analysis revealed no differences regarding myocardial function at follow-up between patients with and without self-reported symptoms (Table 4).

## Discussion

To the best of our knowledge, this is the first study evaluating longitudinal changes in myocardial function in patients with COVID-19 from hospitalization to 4 months after discharge. In this non-selected and consecutively enrolled patient cohort, there was an overall trend towards normalization in myocardial function, predominantly due to a higher rate of normal GLS at follow-up compared to hospitalization. There were no significant changes in LVEF, LV diastolic function and TAPSE over time, but there was a decrease in RV basal diameter. Our findings regarding LV and RV volumes and function during hospitalization for COVID-19 are similar to other echocardiography studies [7, 12, 13] indicating that the absolute values of LVEF, E/e′ ratio, TAPSE, RV S’ are often within the normal range while GLS is reduced [12, 13] during hospitalization. Furthermore, our results are also in line with cardiovascular magnetic resonance imaging studies [14–16] in recovered COVID-19 patients indicating that LV and RV volumes and function are also most often within the normal range in recovered patients. Although the majority of the echocardiographic findings were within the normal range, we did notice a trend towards completely normal TTE’s 4 months after hospitalization, regardless of the biomarkers measured during hospitalization.

Because COVID-19 predominantly affects the respiratory system, theoretically RV function parameters would be expected to show the largest differences between hospitalization and follow-up. The decrease in RV basal diameter at follow-up could hypothetically be due to a decrease or normalization in pulmonary artery pressure after pulmonary recovery, however, pulmonary pressures have not been assessed in the current study. RV S’ was significantly lower at follow-up compared to hospitalization. We considered the change of RV S’ over time as not clinically relevant because the absolute value of RV S’ at follow-up remained within the normal range for all but one patient. The most remarkable difference in functional parameters is seen in GLS. GLS during hospitalization is associated with elevated inflammatory markers and hypoxia suggesting that abnormal GLS could be secondary to systemic inflammation [13]. Three months after COVID-19 inflammatory markers are often within the normal range [17]. Although we did not assess inflammatory markers at follow-up, normalization in inflammatory markers could be a potential cause of the trends towards slight improvement in GLS at follow-up compared to hospitalization.

In large international registries, it is estimated that 27.8% of hospitalized COVID-19 patients require ICU admission [5], 6.6% have pulmonary embolism [5] and that the incidence of elevated Troponin during hospitalization is 23–36% [3, 18]. These findings are comparable with our results. Furthermore, in our and other studies [19, 20], a significant percentage of recovered COVID-19 patients who required hospitalization have self-reported persisting symptoms such as dyspnea, chest pain, fatigue and/or peripheral edema months after discharge. Because of these in-hospital complications and persisting symptoms, cardiac screening for myocardial dysfunction in recovered COVID-19 patients is a matter of debate. Based on our findings, there is no association between elevated cardiac biomarkers, ICU admission, pulmonary embolism nor persisting symptoms, and myocardial dysfunction at follow-up. Therefore, we do not recommend routine cardiac screening with echocardiography in all recovered previously hospitalized COVID-19 patients. Of note, patients with markedly elevated TroponinT, more than three times the upper reference limit of normal, and/or NT-proBNP > 1000 pg/mL during hospitalization are underrepresented in our study and therefore we cannot draw any conclusions from these patients. Further larger scaled studies on cardiac screening and the change in myocardial function in recovered COVID-19 patients are needed.

In conclusion, 4 months after hospitalization for COVID-19, patients had more often a normal TTE than during hospitalization, predominantly related to an increased number of patients with normal GLS at follow-up. There was no association between elevated cardiac biomarkers during hospitalization and myocardial function at follow-up, nor between symptomatology and echocardiographic functional parameters at follow-up.

## Limitations

Our study has limitations. First, the relatively small sample size, and therefore sub-analyses should be carefully interpreted. We tried to include as many patients as possible during the first wave in the Netherlands but due to a drop of hospitalizations in May 2020, we stopped including. Second, since written informed consent was required, the frailest patients could not be enrolled. Third, TTE’s were performed on different ultrasound systems, however, both machines were from the same vendor and analyses were performed using the same software. Fourth, biomarkers were not available at follow-up. Last, we only have follow-up in 40 of the initial 51 patients (78%) [6] which could lead to a selection bias. However, there were no differences in baseline characteristic such as age, sex, elevated TroponinT and/or elevated NT-proBNP between patients who did and did not participate in the follow-up study (supplementary table).

## Supplementary Information

Below is the link to the electronic supplementary material.

Supplementary file1 (DOCX 173 kb)

## Data Availability

The data underlying this article will be shared on reasonable request to the corresponding author.
