# Supplementary material for: Comparison between myocardial function assessed by echocardiography during hospitalization for COVID-19 and at 4 months follow-up
Source: Int J Cardiovasc Imaging. 2021 Jul 20;37(12):3459–67. doi: 10.1007/s10554-021-02346-5 (PMC8294273; doi:10.1007/s10554-021-02346-5)
Supplement: Supplementary file 1 — Supplementary file1 (DOCX 173 kb) [file 10554_2021_2346_MOESM1_ESM.docx]

# Supplemental data

# S1 figure. Comparison of cardiac function parameters in each patient


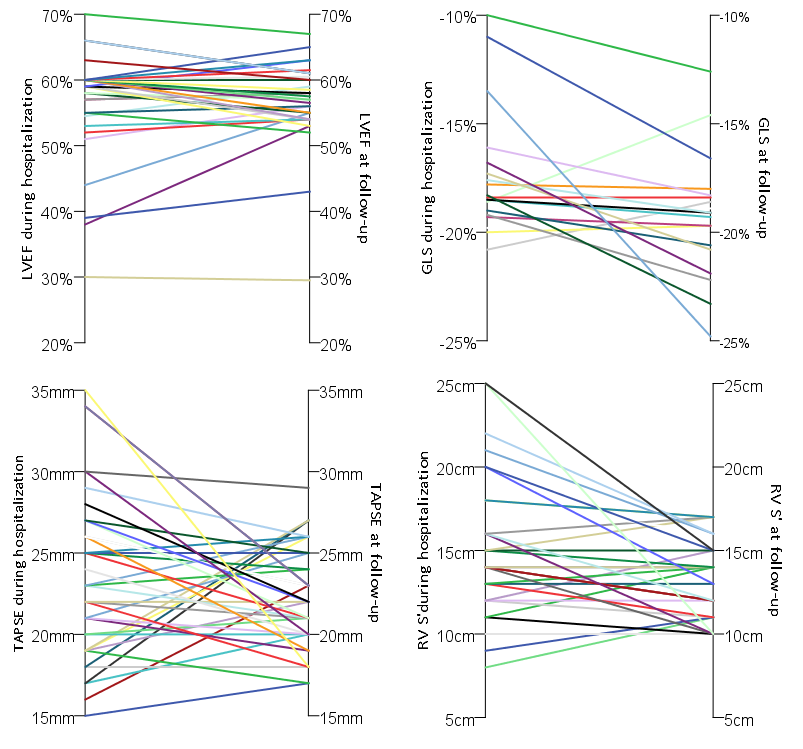


Comparison of left ventricular ejection fraction (LVEF), global longitudinal strain (GLS), right ventricular systolic excursion velocity (RV S’) and tricuspid annular plane systolic (TAPSE) of each patient during hospitalization for corona virus infectious disease 19 (COVID-19) and at follow-up.

# **S2 Table 1.** Comparison between patients who did and did not participate in the follow-up study

|  | **Patients with follow-up**  **N=40** | **Patients without follow-up**  **N=11** | **P value** |
| --- | --- | --- | --- |
| Male | 31 (78%) | 10 (91%) | 0.428 |
| Age (years) | 62.5 (53.5 – 68.0) | 66.0 (36.0 –71.0) | 0.731 |
| Body mass index (kg/m2) | 26.8 (23.7 – 29.0) | 26.8 (25.0 – 29.0) | 0.843 |
|  |  |  |  |
| **Biomarkers** (if available) |  |  |  |
| TroponinT elevated (>14ng/L) | 19 (51%) | 5 (50%) | >0.99 |
| NT-proBNP elevated (>300 pg/mL) | 20 (53%) | 4 (40%) | 0.724 |
|  |  |  |  |

All values were tested with a Wilcoxon signed-rank test. Values are in median and interquartile range, or n (%).
